# Barriers and Facilitators to Implementing the National Patient Safety Implementation Framework in Public Health Facilities in Tamil Nadu: A Qualitative Study

**DOI:** 10.9745/GHSP-D-22-00564

**Published:** 2023-12-22

**Authors:** Yuvaraj Krishnamoorthy, Padmavathi Subbiah, Sathish Rajaa, Murali Krishnan, Krishna Kanth, Gerald Samuel, Isha Sinha

**Affiliations:** a Department of Community Medicine, Employees' State Insurance Corporation Medical College and PGIMSR, Chennai, India.

## Abstract

**Background:**

In 2017, the National Patient Safety Implementation Framework (NPSIF) was introduced in India to ensure patient safety at different levels of the health care delivery system by 2025. Evaluating the implementation status, feasibility, and challenges and obtaining suggestions for improvement are key to the successful and sustainable implementation of any national health framework. Hence, we explored the facilitators and challenges in implementing the NPSIF and sought suggestions to address the challenges.

**Methods:**

We adopted a descriptive qualitative approach to inquire about NPSIF implementation. Health care workers were selected using maximum variability sampling from 18 secondary- and tertiary-level public health care facilities in Tamil Nadu, India. From August to October 2021, we conducted a total of 80 key informant interviews and in-depth interviews with the relevant officers in-charge and HCWs of varied cadres.

**Results:**

Facilitating factors reported were facilities obtaining/working toward quality certification; availability of standard protocols and checklists; and government rewards for the best-performing hospitals, doctors, and staff. Major implementation challenges reported were staff shortages; lack of infrastructure, facilities, and equipment; lack of awareness about patient safety, noncompliance to standard guidelines, and lack of patient cooperation. Recommendations suggested to overcome these challenges included providing educational materials to patients, offering regular continuing medical education and training, improving record maintenance, having a dedicated staff/team and surveillance system setup for patient safety and dedicated staff for data entry, filling existing staff vacancies, and using a carryover option for funding.

**Conclusion:**

Based on the current situation of patient safety practices in public health facilities in Tamil Nadu, it will be difficult to achieve full-scale implementation of the NPSIF by 2025. However, as a first step, a core patient safety committee can be formed at the state level to develop a Gantt chart for implementation based on the priorities over the next 2 years.

## Abstract (precis)

The study findings identified numerous implementation challenges that will make it difficult to fully implement the National Patient Safety Implementation Framework in public health facilities in Tamil Nadu by 2025.

## Teaser

**Key Findings:**

- This study assessing the implementation status of the National Patient Safety Implementation Framework (NPSIF) across 18 public health facilities in Tamil Nadu found that the majority of facilities were still underprepared to implement the patient safety framework in their facilities.
- The findings showed the various challenges in implementing the strategic objectives of the NPSIF, including deficits in infrastructure and staff; lack of maintenance of hospital infection control standards; inadequate biomedical waste management practices; issues around blood safety and medication safety; lack of compliance with standard procedures; and lack of support and funding for patient safety research.
- Recommendations to facilitate implementing the NPSIF included addressing human resource shortages, ensuring adequate infrastructure and logistical support, providing continuous training on standard guidelines and procedures, ensuring compliance through supervision and monitoring, and ensuring support and funding for research.

**Key Implication:**

- A state-level core multidisciplinary expert committee should be formed with subcommittees for each domain of patient safety to discuss and provide recommendations on the implementation of patient safety practices across all public health facilities in Tamil Nadu.

## INTRODUCTION

The World Health Organization (WHO) defines patient safety as the “absence of preventable harm to a patient and reduction of risk of unnecessary harm associated with health care to an acceptable minimum.”^1^ Patient safety represents the “quality” dimension of health care service delivery, an essential component of universal health coverage.^2^ There is a clear consensus that quality health care services across the world should be safe, effective, and people centered. To realize the benefits of quality health care, health services should also be equitable, integrated, timely, and efficient. Implementing patient safety strategies requires having a clear set of policies, employing skilled health care professionals, implementing data-driven safety improvement, and building leadership capacity. Additionally, effective patient involvement in their own care is crucial.^3^

“To err is human,” and expecting a flawless performance from people working in a complex and high-stress environment is very unrealistic. Assuming that individual perfection is possible will not improve the level of patient safety.^4^ Although humans can significantly reduce the likelihood of making mistakes when placed in environments with meticulously designed systems, processes, and tasks, it's important to recognize that no environment can be completely error proof due to the inherent unpredictability and variability of human behavior.^5^ Therefore, efforts should focus on improving health care systems that allow harm to occur. This can only be accomplished in a transparent, open environment where a culture of safety prevails.

Cost-effective precautions, such as hand hygiene, proper waste disposal, personal protective measures, clean hospital environments, and patient etiquette, can ensure patient safety and reduce the burden of health care-associated infections and other adverse events associated with patient safety.^6^ Therefore, investments are needed that would enable hospitals and health care facilities to be safe for patients. Making health care facilities safe could also substantially reduce associated health care costs and improve patient satisfaction and experiences. Recognizing the importance of these precautions, the India Ministry of Health and Family Welfare introduced the National Patient Safety Implementation Framework (NPSIF) in 2017. The framework aims to improve patient safety at all levels of the health care delivery system in both public and private facilities across all modalities of health care provision, including the prevention, diagnosis, treatment, and follow-up within the overall context of improving the quality of care and progressing toward universal health coverage by 2025.^7^

The NPSIF was introduced in 2017 to ensure patient safety at different levels of the health care delivery system in both public and private facilities across India.

**FIGURE 1:**
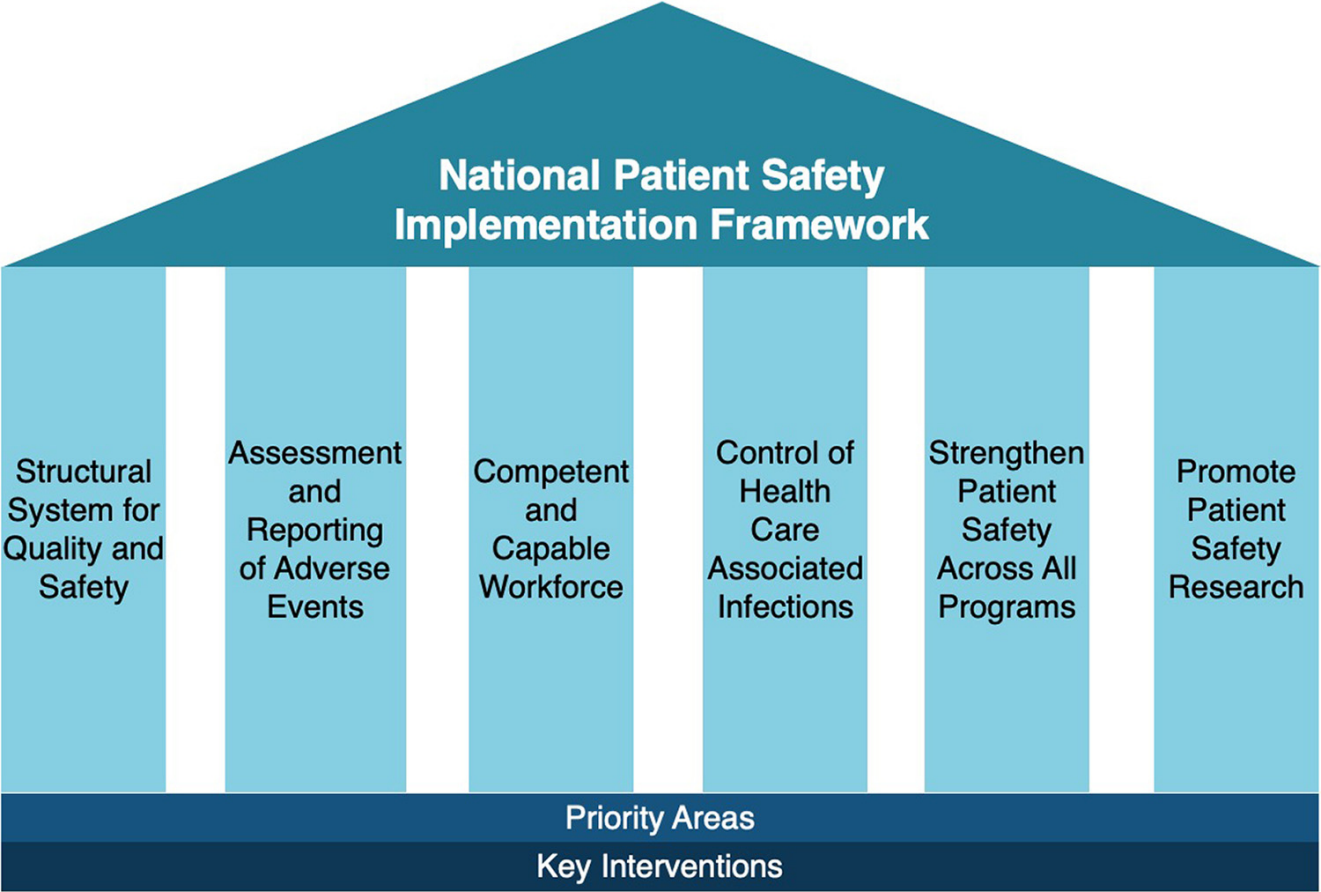
Strategic Objectives of the National Patient Safety Implementation Framework, India Source: Government of India. Ministry of Health and Family Welfare 2018.^7^

The National Patient Safety Steering Committee was vested with the responsibility of implementing the NPSIF under the aegis of the Ministry of Health and Family Welfare. Each of the 6 strategic objectives outlined in the NPSIF includes a timeline, responsible organizations, priority areas (short-, mid-, and long-term), and expected outputs (Figure 1). The framework also required additional human resources, funding from the central and state governments, and efficient utilization of the existing infrastructure available for quality assurance and vertical national health programs. A monitoring and evaluation plan with priority indicators was also provided.

However, the strategies, action plan, funding requirements, and monitoring were primarily given as recommendations, and the status of actual implementation has not been explored much in the country. Evaluating the implementation status, feasibility, and challenges and seeking improvement suggestions are key to the successful and sustainable implementation of any national health programs, schemes, or frameworks. Hence, we have explored the facilitators and challenges in implementing the NPSIF and sought suggestions to overcome these challenges through qualitative interviews.

## METHODS

### Study Design

This study was conducted as part of a large-scale sequential explanatory mixed method study comprising a quantitative process evaluation of the NPSIF^8^ and a qualitative component with key informant interviews and in-depth interviews of health care workers (HCWs) and officials in the surveyed facilities. We adopted a descriptive qualitative approach to inquire about NPSIF implementation in terms of facilitating factors, barriers, and suggestions.

### Study Setting and Sampling

NPSIF is mainly focused on secondary and tertiary care facilities in the country. Hence, we only sampled these 2 levels of health care facilities in Tamil Nadu, a state in the south of India. We performed a 2-stage stratified random sampling to select the public health facilities.

In the first stage, all 6 districts were stratified into 3 categories (low, medium, and high) based on their Human Development Index scores. We chose this method to stratify the districts because the indicators in the Human Development Index were representative of important demand-side characteristics explaining the health status, health care-seeking behavior, and utilization of services. Two districts from each of these strata were selected randomly using the lottery method.

In the second stage, we selected a total of 18 secondary- and tertiary-level public health facilities from the 6 districts: 6 tertiary care hospitals in each selected district and 2 government hospitals from each district.

### Participants

Key informant interviews and in-depth interviews were conducted with the relevant officers in-charge (e.g., dean, medical superintendent, or resident medical officer), respective committee heads, and HCWs of varied experience and cadres (Table).

**TABLE.** Characteristics of Participants Interviewed About Implementation of the National Patient Safety Implementation Framework in Tamil Nadu, India

	No. (%) (N=80)
Gender	
Male	27 (33.7)
Female	53 (66.3)
Health care worker cadre	
Doctor	44 (55.0)
Nurse	34 (42.5)
Other allied staff (pharmacists, health management information system staff)	2 (2.5)
Job position	
Dean, medical superintendent, resident medical officer, nursing superintendent	32 (40.0)
Head or staff under hospital infection control committee, central sterile supplies department, biomedical waste, microbiology department	26 (32.5)
Head or staff under transfusion committee/blood bank/immunohematology	7 (8.8)
Head or staff under pharmacovigilance committee/pharmacology department	9 (11.3)
COVID-19 nodal officer	4 (5.0)
Head or staff under antimicrobial stewardship committee	1 (1.2)
Obstetrics and gynecology professor in charge of Labour Room Quality Improvement Initiative and health management information system	1 (1.2)

### Data Collection

At least 5–6 in-depth interviews were conducted per facility between August and October 2021. The participants were purposively selected through maximum variation sampling across different departments, committees, cadres, and experiences at different levels of care. After conducting the quantitative survey and analyzing its findings, the principal investigator developed and refined an interview guide. This guide was employed to gather insights about the facilitators and challenges encountered in implementing the NPSIF across all strategic objectives. Furthermore, participants were invited to share their recommendations for addressing these challenges.

Interviews were conducted by a trained set of data collectors who were fluent in the local language, formally trained in a qualitative research workshop, and had previous experience in the qualitative data collection process. They were trained on the study methodology by the investigators.

Interviews began after interviewees gave their informed consent and were instructed on the purpose and motive of the study. The interviews lasted 45–60 minutes. Privacy was ensured by conducting the interview in an isolated room without the presence of nonparticipants. Participants were ensured of the confidentiality of the information obtained through the interview. The interviews were audio-recorded only for participants who provided consent. Field notes were taken during the interview.

At the end of the interview, a summary was presented to the participants for validation of the data. Data triangulation was done through multiple sources. To ensure methodological rigor, we physically verified the records and meeting minutes of the hospital infection control committee (HICC) and biomedical waste (BMW) management committee, registers of the central sterile supplies department and blood bank, reports of adverse drug reactions (ADRs), and inventory logs of medicines and supplies.

### Analysis

Interviews were transcribed verbatim within the same day of the interview (to prevent the loss of information). Transcripts were reviewed by another person to decrease the bias and increase the interpretive credibility. Manual thematic content analysis was performed to derive the themes and subthemes. We derived codes using a hybrid approach (inductive and deductive). The findings were reported by using consolidated criteria for reporting qualitative research.^9^

### Ethical Approval

This study was approved by Institutional Ethics Committee of ESIC Medical College & PGIMSR, Chennai, April 5, 2021, with IEC No. IEC/2021/1/12.

## RESULTS

In total, 80 qualitative interviews were conducted with administrative heads and HCWs across 18 public health care facilities in Tamil Nadu. The mean age of all participants was 48 years (Table).

We used 3 predetermined themes (facilitating factors, challenges, and suggestions for overcoming challenges) with 9 subthemes (structural system for quality and safety, hospital infection control, BMW management, blood safety, antimicrobial stewardship, COVID-19 safety, medication safety, procedural and device safety, and patient safety research). Subthemes were incorporated from the strategic objectives of the NPSIF, while the themes were developed during the data collection process (Supplement Tables S1–S3).

### Subtheme 1: Structural System for Quality and Safety

Positive patient feedback served as a significant motivator for hospitals working toward accreditation. Conversely, poor patient cooperation emerged as a major barrier. Motivated HCWs with multitasking skills and a sense of reputation facilitated progress at the individual level. Yet, many HCWs felt overburdened by clerical tasks linked with the certification process.

Many HCWs felt overburdened by clerical tasks linked with the quality certification process.

*We are overloaded with clerical works; we are not able to give our 100% to patient care.* —HCW, secondary-level facility

At the hospital administration level, various initiatives had been introduced, including staff training in the Labour Room Quality Improvement Initiative (LAQSHYA), strategic placement of complaint boxes, and the establishment of a patient welfare committee. However, the hospital administration also faced challenges, including outdated infrastructure, insufficient facilities, and financial constraints.

*We have enough funds but carry over option would be a better option; we can perform better when the funds don't come with expiring date.* —Medical superintendent, tertiary-level facility

Training specifically designed for the accreditation process was seen as crucial at the health system level, but challenges such as staff rotations after training persisted.

*We have received LAQSHYA certificate but sustaining it becomes challenging especially with shortage of staffs. Nurse whom we train here for a good time by the time they learn properly they get rotated and it becomes difficult for us.* —LAQSHYA in-charge, tertiary-level facility

### Subtheme 2: Hospital Infection Control

The COVID-19 pandemic heightened the importance of hospital infection control.

*COVID-19 fear led to patients realizing importance of hand hygiene.* —Nursing superintendent, tertiary-level facility

However, factors such as crowding and patient noncompliance impeded the maintenance of high standards of infection control.

*Patient cooperation is must; people get crowded inside ICU.* —Head of HICC, tertiary-level facility

HCWs found motivation through public recognition for infection control and the facility's recognition for receiving the Kayakalp quality certification. Yet, adapting to frequent updates in protocols/guidelines and finding time to review records remained significant challenges.

*Suddenly, some new guidelines come up and we have to adapt immediately to that protocol; this is difficult especially during first few weeks.* —HCW, tertiary-level facility

Hospital administrations undertook hospital infection control initiatives, such as disseminating information through educational materials and digital data management. The introduction of innovative cleaning practices, such as the “3 bucket system,” showcased adaptability.

*We use 3 bucket system for ensuring a clean surface; zig-zag method of mopping is taught to our sanitary workers.* —HCW, tertiary-level facility

However, the ongoing COVID-19 crisis and lack of staff posed challenges to routine infection control activities.

### Subtheme 3: BMW Management

Overcrowding and littering behavior remained major challenges for proper BMW management. Within the health care sector, there was resistance among HCWs to attend the BMW management training and a general noncompliance with standard guidelines.

*They are not interested or overworked we put training for 10 people but 4 turn up.* —Head of HICC, tertiary-level facility

Proactive measures at the hospital administration level included regular audits, monitoring, and rewards for staff following best practices. Yet, challenges persisted, such as the absence of a sewage treatment plant and irregular inspection of BMW disposal practices.

*We are already overburdened with so much administrative work; we don't have enough staffs to allot for monitoring the biomedical waste disposal in our hospital.* —Medical superintendent, secondary-level facility

At the health system level, there was a significant challenge in securing a consistent supply of essential BMW management materials.

### Subtheme 4: Blood Safety

Community support for blood donation camps was a positive factor at the patient level. However, issues with donors involved in high-risk behaviors and professional donors presented challenges.

*There are professional donors who sell blood for money, people still fear for blood donation they go for professional donors.* —Head of transfusion committee, tertiary-level facility

Hospital administrations focused on patient representation, awareness programs, and leveraging technology to ensure blood safety. However, the COVID-19 pandemic posed challenges, notably in organizing blood donation camps.

*Because of COVID blood donation camps are not being conducted, so we have to go for replacement donors.* —Head of transfusion committee, tertiary-level facility

### Subtheme 5: Antimicrobial Stewardship

Patient self-medication with higher-level antibiotics emerged as a significant issue. Furthermore, some HCWs tended not to adhere to the antibiotic prescription guidelines set by the Indian Council of Medical Research.

*Some patients do not follow doctor's advice and take some high-end antibiotics from their local pharmacy if the symptom persists for even 2 days.* —Head of HICC, tertiary-level facility

*Some of them (doctors) think higher antibiotics will work and ask the patients to take from local purchase.* —Head of HICC, tertiary-level facility

One solution was the crosschecking of prescriptions for higher-level antibiotics by respective department heads. Challenges within the hospital administration included frequent stock-outs of antimicrobials and lack of prescription audits.

### Subtheme 6: COVID-19 Safety

Patients generally appreciated HCWs' efforts during the pandemic and were willing to comply with safety measures. However, issues arose from patients' unwillingness to undergo triaging or leaving isolation against medical advice.

*COVID positive patients are not staying in hospital and going back to home without listening to our orders and come back whenever they feel like.* —Chief medical officer, secondary-level facility

At the health system level, measures such as outsourcing and psychological support for patients were implemented. However, the lack of dedicated staff for COVID-19 duties remained a challenge.

### Subtheme 7: Medication Safety

Challenges at the patient level included discontinuation of medications after experiencing an ADR without consulting a doctor.

*Because of ignorance they (patients) will not know that the problem they are facing is because of the drug (they take) and they will be taking alternate medicines or alternate medical treatment.* —Head of pharmacovigilance committee, tertiary-level facility

HCWs sometimes exhibited resistance to standard protocols, and timely reporting of nearly expired drugs was a concern.

*Doctors are not willing to follow the standard guideline as they follow their own clinical practice.* —Head of pharmacovigilance committee, tertiary-level facility

Hospital administrations focused on regular audits and training sessions for handling ADRs, but there remained a gap in awareness of reporting them in some hospitals.

*We neither did not know that we need to report adverse reactions nor procedure to do.* —Medical officer, secondary-level facility

### Subtheme 8: Procedural and Device Safety

Patients expressed satisfaction after delivering in health facilities certified by the LAQSHYA. However, patient demand for unnecessary treatments and procedures was a concern.

*Patients are obsessed in demanding for injection and CT scan.* —HCW, secondary-level facility

Among HCWs, the lack of compliance with standard guidelines posed a challenge. Meanwhile, hospital administration initiatives focused on training and introducing standard protocols, though there were infrastructural and staffing challenges.

*[Central sterile supplies department] staffs are rotated to other departments after training; this makes us to give excess undue training to newer staffs; monitoring can happen effectively if they are kept in 1 place.* —Head of microbiology department, tertiary-level facility

### Subtheme 9: Patient Safety Research

Patients' participation in satisfaction surveys facilitated quality monitoring. Although faculty in medical colleges were motivated for research, challenges included a lack of awareness and support for research on patient safety.

*We are unaware about the theme “patient safety” and hence we have not undertaken research on this topic.* —Medical superintendent, tertiary-level facility

Most of the surveyed facilities were using some best or innovative practices that could be adopted across all the secondary and tertiary care facilities in Tamil Nadu (Supplement Table S4).

## DISCUSSION

**FIGURE 2:**
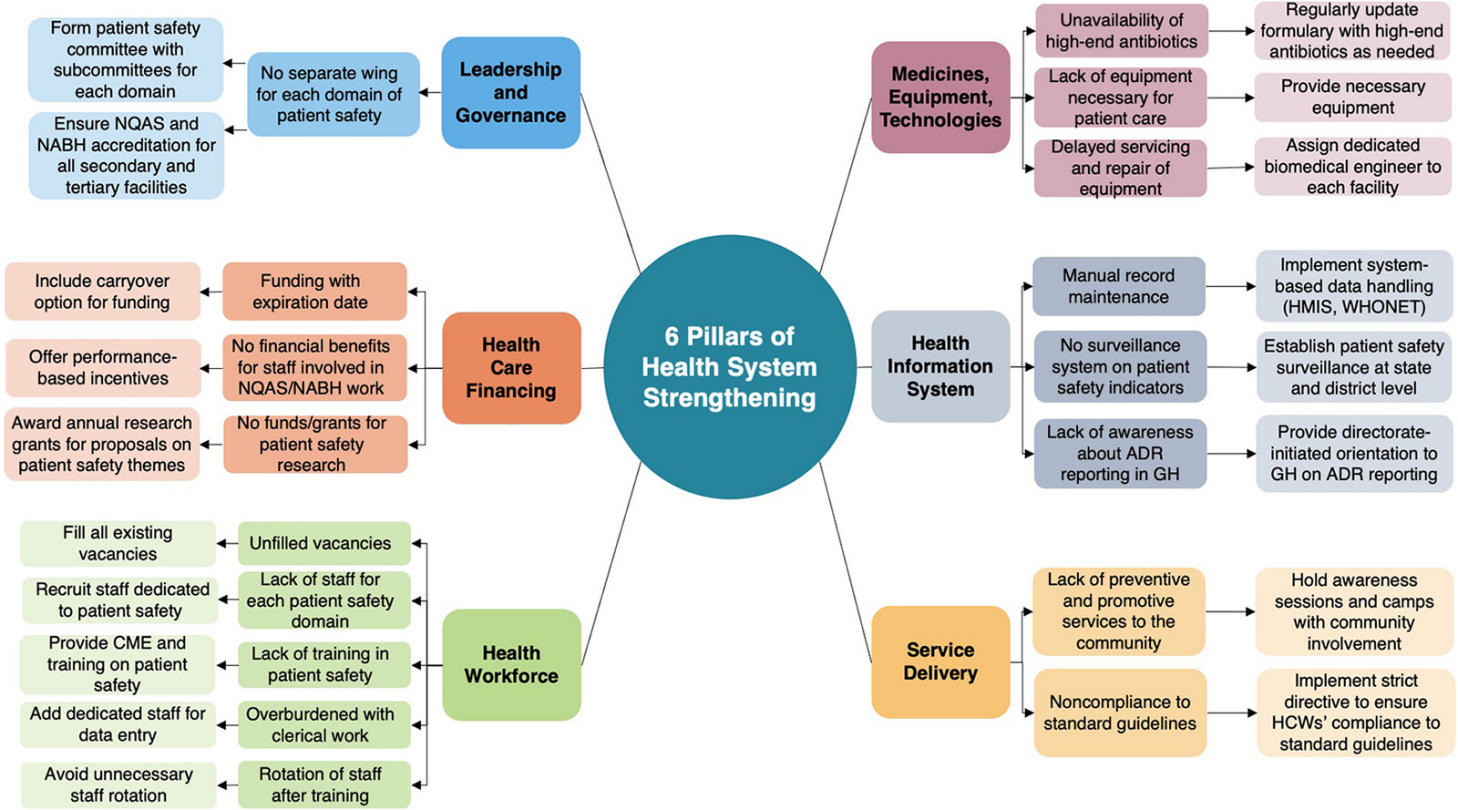
Conceptual Framework^a^ of Challenges and Recommendations for Application of the National Patient Safety Implementation Framework Across Public Health Facilities in Tamil Nadu Abbreviations: ADR, adverse drug reaction; CME, continuous medical education; GH, government hospital; HCW, health care worker; HMIS, health management information system; NABH, National Accreditation Board for Hospitals and Healthcare Providers; NQAS, National Quality Assurance Standards. ^a^ Based on the World Health Organization's 6 pillars of health system strengthening.^10^

Our study shows the lack of readiness and various challenges in implementing the activities mentioned under the NPSIF across all the surveyed public health facilities in Tamil Nadu. We developed a conceptual framework based on WHO's 6 building blocks of health system strengthening^10^ to depict the major challenges and recommendations reported during the qualitative interviews (Figure 2). We also assessed the facilitating factors, challenges, and recommendations under the following subthemes.

### Structural System for Quality and Safety

The achievement of the NPSIF remains challenged, primarily due to deficits in the structural system for quality and safety. A significant proportion of health care facilities failed to attain accreditation, with key impediments being infrastructure, staffing, equipment, and technological limitations, compounded by financial constraints and excessive demands on HCWs. This observation aligns with a systematic review assessing stakeholder attitudes toward the accreditation process, which cited reasons akin to our study, such as financial barriers and demanding staff, as primary culprits for not attaining accreditation in various facilities.^11^

The achievement of the NPSIF remains challenged, primarily due to deficits in the structural system for quality and safety.

To navigate these hurdles, we recommend a multipronged approach. Addressing human resource shortages through filling existing vacancies and hiring new staff specifically for quality control emerged as a priority. Further bolstering these efforts should be the incentivization of staff, meticulous recordkeeping through logs and registers, and the promotion of continuous medical education through workshops and training on the latest guidelines. Filling pivotal administrative roles—such as dean, medical superintendent, and resident medical officer—with experienced professionals who have a background in hospital administration is another crucial step.

The broader health system should consider establishing distinct wings for each patient safety domain at the directorate level. Integrating quality and patient safety into the curriculum of medical, dental, and allied health disciplines ensures future professionals prioritize these concerns. Accreditation standards and award criteria should more prominently feature patient safety.^6^ While the latest iteration of the National Quality Assurance Standards accreditation process incorporates a component of patient safety, it is vital that it fully encompasses all aspects outlined in the NPSIF.^12^ For facilities aiming for global benchmarks, addressing patient safety requisites via international standards like the Joint Commission International certifications may be beneficial.^13^

### Hospital Infection Control Policies

The ongoing COVID-19 crisis actually had a positive impact on infection control measures, as most of the public health facilities conducted training for HCWs on hand hygiene and personal protective equipment use and installed handwashing stations with sanitizers and awareness sessions for the patients.

Compliance with these practices was also heavily scrutinized during this period. This showed that the public health facilities in Tamil Nadu were working in a positive direction for handling infection control practices. However, the sustainability of these measures is important, and a future survey on the same set of indicators after the pandemic could indicate hospital infection control practices in normal situations.

### BMW Management Policies

The ongoing COVID-19 crisis led to a positive change in waste disposal practices, as most facilities conducted training with HCWs on BMW disposal practices and management. However, patient noncompliance with proper waste disposal practices was an important issue, as the general public, patients, and their families also started using a wide range of personal protective equipment, which was considered BMW.^14^

Patient engagement and institutional support emerged as 2 key pillars in ensuring effective BMW management. At the patient level, there was a clear shift toward fostering a sense of collective responsibility and ownership. The “no waste littering policy” aimed to instill discipline and awareness about waste disposal, and the “our hospital” concept was an innovative approach that reframed the hospital space as a shared, communal asset. By viewing the hospital as “ours,” patients were more likely to actively participate in maintaining its cleanliness and upholding the standards.

Conversely, at the health system level, the foundation of effective BMW management rested on logistical support. Ensuring an uninterrupted supply of essential management materials—from basic items like buckets, covers, and gloves to more specialized equipment like closed bins for trolleys—was paramount. These tangible resources not only facilitated the proper segregation and disposal of waste but also underscored the system's commitment to safeguarding both patient and environmental health.

### COVID-19 Safety Policies

Although COVID-19 safety was not a component of NPSIF (given the nonexistence of the disease during the period of framework development), the global patient safety action plan emphasized that the ongoing pandemic elicited some important patient safety implications, giving a heightened impetus to the efforts that promoted safe care at every level.^6^

The shared commitment and responsibility united the stakeholders and HCWs like never before. Many facilities spontaneously adopted some key safety attributes such as collaboration, active communication, transparency, and rapid adoption of newer patient safety practices (e.g., implementing new hand hygiene strategies and facilities). However, representatives of these facilities responded that they did not want them to only be temporary measures but to be sustained and further strengthened. The forthcoming years will be time to build a safer health system that minimizes avoidable harm to patients and HCWs. Contemplating how the COVID-19 situation adds to the patient safety context will help in gleaning newer patient safety lessons from both the pandemic failures and transformations.^15^

### Medication Safety Policies

Medication safety was deeply intertwined with levels of engagement from patients to health systems. At the patient level, health literacy stood out as a fundamental component of medication safety. It was not just about reading medical jargon but understanding and acting on it, especially concerning ADRs. The emphasis on the role of HCWs in educating about ADRs was further validated by the insights of a pharmacology expert, underlining the necessity of patient education during prescription.

At the broader levels of hospital administration and health systems, the approach to medication safety expanded. Celebrating “pharmacovigilance week,” for example, showcased a dedication to creating awareness, not just among patients but within the institutional framework. Additionally, systems needed to prioritize infrastructural consistency, for example, by ensuring antibiotic availability across care facilities and establishing surveillance for even minor ADRs. Such strategies, when viewed holistically, set the foundation for enhanced medication safety and overall patient care. A best practice adopted by a surveyed facility followed the “10R” checklist for safe drug administration (“right patient, right reason, right drug, right route, right time, right dose, right form, right action, right documentation, and right response”).^16^ Adoption of this checklist advocated the need for knowledge of the causes of medication errors, the process of implementing strategies to reduce medication errors, and the use of safe practices throughout the medication journey.

### Blood Safety Policies

Patient engagement and representation emerged as pivotal facets of health care quality, especially in specialized areas like blood transfusion. The recommendation at the patient level stressed the inclusion of patients in the blood transfusion committee. Such a move was not just a token of inclusivity; it brought a unique perspective of ensuring that policies were developed with patient interests at the forefront. The assertion from the head of the transfusion committee echoed this sentiment, emphasizing the potential of patient-friendly policies born of such collaborations. Furthermore, the analogy with organ donation highlighted a poignant aspect of blood transfusion—the invisible bond between the donor and the recipient.

At the hospital administration level, 2 key recommendations emerged. First, ensuring that blood donors received acknowledgment and appreciation became a significant morale booster, creating a positive feedback loop encouraging further donations. Second, ensuring medico-legal safety for blood handlers underpinned the delicate nature of blood transfusion, highlighting the importance of protecting those providing this essential service. Both recommendations, when synthesized, presented a holistic approach to transfusion safety, intertwining patient interests, donor recognition, and staff protection.

Professional donors selling blood for monetary benefits was reported as a major challenge. The Supreme Court of India already mandated the removal of professional blood donation.^17^^,^^18^ However, as per study findings, it was clear that this practice still occurred. In addition, the practice of using replacement donors was still present in most facilities. WHO reported that the elimination of the family/replacement donor and paid donor system was a key component in establishing a safe blood transfusion service.^19^ Hence, it is important to further eliminate these practices and promote nonremunerated voluntary blood donation practices across all the public health facilities in Tamil Nadu. However, the trend in voluntary blood donation practice worsened due to the ongoing COVID-19 crisis.

### Antimicrobial Policies

The escalating challenge of antimicrobial resistance underscored the dire need for robust antimicrobial stewardship measures across various levels of health care. For HCWs, we propose a tangible approach to gauge antibiotic use by using the pill count method during patients' follow-up visits. This technique offers a real-time assessment, ensuring the appropriateness of antibiotic usage and potentially curtailing overuse or misuse.

In analyzing hospital administration directives, a multipronged strategy emerges. First, sending clear directives to all clinical departments emphasizes a unified approach toward judicious antibiotic application. Second, regularly updating the antibiotic formulary ensures that prescriptions remain aligned with evolving microbial patterns and resistance profiles. Further emphasizing consistent training for antibiotic prescriptions underscores the dynamic nature of antimicrobial stewardship and the need for continuous education.

From a broader health system perspective, innovation and integration play pivotal roles. Digitally monitoring antibiotic usage offers a real-time, data-driven approach, ensuring oversight and facilitating corrective actions. The proposition of a dedicated wing of experts to oversee this initiative underscores its importance. Equally compelling is the recommendation to embed antimicrobial stewardship principles within the curriculum of medical, dental, and allied health sciences.

### Procedural and Device Safety Policies

Major challenges faced across these facilities were patients' obsessions with unnecessary treatment and procedures (injections and scans); noncompliance of HCWs to standard guidelines; and lack of staff, infrastructure, and equipment. Procedural and device safety were quintessential facets of a hospital administration's commitment to ensuring the highest quality of care. Central to this commitment was the implementation of standardized protocols, as exemplified by the safe surgical checklist. Such checklists not only standardize procedure but also serve as a safety net, catching potential oversights before they culminate in clinical errors. Moreover, the importance of equipment reliability cannot be overstated. Regular calibration of essential tools, like the electronic blood pressure apparatus, and consistent annual maintenance of various equipment underscores the pivotal role of technology in patient safety. Beyond the machinery, fostering an environment of continuous learning is paramount. Clinical society meetings offer a platform for physicians across specialties to share, learn, and deliberate on challenges faced during major procedures, thus ensuring that the collective wisdom of the medical community is harnessed to continually enhance patient care.

Implementing standardized protocols, maintaining equipment reliability, and fostering an environment of continuous learning are all key to ensuring the highest quality of patient care.

### Patient Safety Research

Fostering patient safety research requires a significant effort to strengthen the research capacity, as the underdevelopment of this research was attributed to a lack of support, funding, and encouragement at the administration and health system levels. Hence, it is important to encourage and motivate researchers in public health facilities about the primary purpose of patient safety research and provide appropriate funding mechanisms. Doing this will enable the production of interventions and solutions for safer patient safety policies and practices.^20^ It is necessary to develop a set of professional leaders who will be able to drive the change through monitoring and research.^21^

The inclusion of patient safety into the curriculum might help to encourage more professionals to focus on this area of research. WHO released a guide for developing training programs on patient safety research that helps to build the capacity of leaders in patient safety research, implementation, and change management.^22^ This is especially important in a setting like India where the need for cost-effective and locally acceptable interventions is more critical.^23^^,^^24^

### Findings in the Context of High-Reliability Organizations

High-reliability organizations operate in complex and high-risk environments but consistently achieve high levels of safety and reliability.^25^ In the context of health care, high-reliability organizations are particularly relevant, as health care facilities are also high-risk environments where errors and adverse events can have serious consequences for patients.

Weick and Sutcliffe provide valuable insights on how organizations can achieve high reliability in complex and dynamic environments.^26^ Three important points from their work are relevant to our study.

- “Preoccupation with failure” involves a mindset where organizations are constantly anticipating and preparing for potential failures or errors. In the context of our study, this could translate to hospitals and health care systems being proactive in identifying and addressing potential challenges and barriers to patient safety and quality of care.
- “Sensitivity to operations” refers to the ability of organizations to identify and respond to unexpected changes or deviations from normal operations. In the context of our study, this could mean hospitals and health care systems have a robust system for monitoring and addressing infection control, blood safety, and antimicrobial stewardship practices.
- “Reluctance to simplify” involves recognizing the complexity and interconnectedness of various factors that contribute to safety and reliability and avoiding oversimplification or reductionism. In the context of our study, this could mean acknowledging and addressing the multifaceted challenges and barriers to achieving high reliability in health care settings, such as lack of infrastructure, inadequate staffing, and poor supply of materials.

We believe that these 3 points highlight the importance of a proactive and vigilant approach to safety and reliability in health care settings. By applying these principles, hospitals and health care systems can work toward becoming high-reliability organizations and improve patient safety and quality of care.

## RECOMMENDATIONS

- Form a state-level core multidisciplinary expert committee with subcommittees for each domain of patient safety to provide recommendations on implementing patient safety practices across all public health facilities in Tamil Nadu. The committee would assign the directives and standards for implementation at the facility level and monitor facilities by reviewing their reports and undertaking audits and assessments.
- Establish state- and district-level surveillance of patient safety practices and implementation of the patient safety framework and its outcomes at nodal centers and several smaller regional centers.
- Create a statutory requirement and accountability mechanism for all public health facilities to operate in a transparent manner, ensure minimum patient safety standards, and publish an annual report on patient safety.
- Establish a state- and district-level patient safety charter that includes the institutional standards and the rights and responsibilities of patients and HCWs for various patient safety domains.
- Have district quality control teams conduct periodic audits of all the public health facilities under their control to identify the required infrastructure, facility, and equipment for achieving the National Quality Assurance Standards and National Accreditation Board for Hospitals and Healthcare Providers accreditation process.
- Establish patient safety advocates and champions networks at facility, district, and state levels. This network should consist of patients and families that have experienced adverse events in hospitals to use their experience of safe and unsafe care positively and build safety and harm-minimization strategies. Patients and their families can be involved at all possible levels of health care delivery, ranging from the policymaking and planning related to patient safety to performance oversight and shared decision-making at each level of care. However, the patients need to be educated first as involvement before proper education can result in conflict within the health care facilities.

### Study Strengths

This was the first study to assess the implementation of the patient safety framework in public health facilities in India. Data triangulation was done through multiple sources, and the information obtained was physically verified through records, registers, or logs. Triangulation and respondent validation further enhance the credibility of the study findings. The use of maximum variability sampling by involving relevant stakeholders and a detailed description of study findings may help researchers decide on the transferability of findings across contexts.

### Limitations

Despite its strengths, the study has certain limitations. There is a possibility of desirability bias, as the HCWs might tend to under-report the unfavorable findings and over-report the favorable information about their own health facility. Qualitative interviews with state- and district-level policymakers were not conducted to understand the feasibility of implementation of the proposed recommendations and the challenges to implementing the NPSIF at state and district administration levels. We have included only secondary and tertiary care health facilities, as the NPSIF focuses primarily on the higher level of care. However, patient safety is a universal concept applicable at all levels of health care in both private and public facilities. Hence, future studies should also include primary health care facilities and private health care facilities.

## CONCLUSION

The study reveals that the current situation of patient safety practices in public health facilities is not conducive to a full-scale implementation of the NPSIF by 2025. The findings suggest that there are significant barriers that need to be addressed, such as inadequate resources, lack of training and awareness among health care providers, and weak regulatory mechanisms. However, the study also identifies a potential way forward. By forming a core patient safety committee at the state level and developing a Gantt chart for framework implementation based on priorities over the next 3 years, health care providers and policymakers can take a step toward improving patient safety in public health facilities.

Overall, the study highlights the importance of addressing patient safety as a critical aspect of quality of care. Although there are significant challenges in implementing the patient safety framework in public health facilities in India, concerted efforts by all stakeholders can help overcome these barriers and pave the way toward improving the quality of care for patients.

## Supplementary Material

GHSP-D-22-00564-supplement.pdf

## Notes

**Peer Reviewed**

**First published online:** November 3, 2023.
