# Supplementary material for: Barriers and Facilitators to Implementing the National Patient Safety Implementation Framework in Public Health Facilities in Tamil Nadu: A Qualitative Study
Source: Glob Health Sci Pract. 2023 Dec 22;11(6):e2200564. doi: 10.9745/GHSP-D-22-00564 (PMC10749659; doi:10.9745/GHSP-D-22-00564)
Supplement: GHSP-D-22-00564-supplement.pdf [file GHSP-D-22-00564-supplement.pdf]

**Theme: Facilitating factors for implementation of National Patient Safety Implementation Framework**		
**Subthemes**	**Categories**	**Codes**
**Structural System for Quality & Safety**	**Patient**	➢ Positive feedback from patients
	**Healthcare workers**	➢ Motivated workforce with multi-tasking ability<br>➢ Sense of reputation and identity
	**Hospital Administration**	➢ LAQSHYA training<br>➢ Patient welfare committee<br>➢ Compliant boxes/Anonymous reporting system<br>➢ Infant monitoring system
	**Health System**	➢ Training for NQAS accreditation
**Hospital Infection Control**	**Patient**	➢ Fear/Threat of COVID-19
	**Healthcare workers**	➢ Job Satisfaction for performing public service<br>➢ Teamwork and supportive supervision
	**Hospital Administration**	➢ COVID-19 crisis led improvement in hand hygiene & PPE training<br>➢ Widespread depiction of IEC materials<br>➢ Regular HICC meeting with rapid problem solving<br>➢ Innovative cleaning practices<br>➢ Utilization of “WHO NET software”<br>➢ Work sampling & observations for monitoring the hand hygiene practices

	**Health System**	➢ Standard protocol for hospital infection prevention & control measures
**Biomedical Waste Management**	**Patient**	➢ Understanding the hazards of biomedical wastes
	**Healthcare workers**	➢ Sense of responsibility & contribution
	**Hospital Administration**	➢ Regular audits and practice sessions<br>➢ Appraisal, appreciation, and rewards<br>➢ Work sampling to monitor BMW practices
**Blood Safety**	**Patient**	➢ Local community support in blood donation camps
	**Healthcare workers**	➢ Job satisfaction for saving patient's lives<br>➢ Counselling for blood donation<br>➢ Commitment of healthcare workers
	**Hospital Administration**	Patient representation to frame blood safety policy<br>➢ Awareness program on blood donation practices<br>➢ Application of latest technologies (Gel card technique, apheresis)
	**Health System**	➢ Political support for blood donation camps
**Antimicrobial Stewardship**	**Healthcare workers**	➢ Counterchecking prescription of high-level antibiotics
	**Hospital Administration**	➢ Monitoring antibiotic practices as part of accreditation process<br>➢ Regular CME, Seminars & Case discussions
	**Health System**	➢ Directorate-initiated appropriate antibiotic use training for HCWs
**COVID-19 Safety**	**Patient**	➢ Willingness to comply COVID-19 measures

		➢ Appreciating HCWs for doing COVID-19 duty
	**Healthcare workers**	➢ Sense of responsibility, satisfaction, selfless service to the community
	**Hospital Administration**	➢ Security personnel monitoring visitors for COVID appropriate behaviour<br>➢ Triage system to identify COVID-19 suspects<br>➢ Quarantine for HCWs as per protocol
	**Health System**	➢ Support in outsourcing manpower & equipment<br>➢ Advanced IEC materials for awareness sessions<br>➢ Mobile phones for patients to communicate with their family
**Medication Safety**	**Healthcare workers**	➢ Interest in undertaking pharmacogenomic studies
	**Hospital Administration**	➢ Regular meeting and audits of pharmacovigilance committee<br>➢ Regular training for handling emergency ADR<br>➢ "10R" checklist for drug administration
	**Health System**	➢ Clinical pharmacology wings at district level
**Procedural and Device Safety**	**Patient**	➢ Satisfaction among delivered mothers in LAQSHYA certified facilities
	**Hospital Administration**	➢ Recruitment and refresher training on safe procedural practices<br>➢ Mercury spill kit and training<br>➢ Standardized protocol for major procedures<br>➢ Knowledge about standardized procedures through accreditation process

**Patient Safety Research**	**Patient**	➢ Patients Cooperation
	**Healthcare workers**	➢ Motivated Faculties in medical colleges to conduct patient safety research

Theme: Challenges in implementation of National Patient Safety Implementation Framework		
**Subthemes**	**Categories**	**Codes**
**Structural System for Quality & Safety**	**Patient**	➢ Poor patient cooperation
	**Healthcare workers**	➢ Overburdened with clerical works
	**Hospital Administration**	➢ Lack of infrastructure necessary for accreditation<br>➢ Lack of equipment necessary for accreditation<br>➢ Shortage of manpower necessary for accreditation<br>➢ Fund spending within deadline<br>➢ No structural/technological improvement<br>➢ No stress management classes for staffs
	**Health System**	➢ Rotation of staffs between facilities<br>➢ Unfilled vacancies of various posts<br>➢ Delay in funding
**Hospital Infection Control**	**Patient**	➢ Overcrowding and uncooperative patients
	**Healthcare workers**	➢ Difficulty in adapting to newer protocols<br>➢ No time to review records and logs<br>➢ HICC staffs unaware of job responsibilities

	**Hospital Administration**	➢ COVID-19 crisis affects HICC coordination
	**Health System**	➢ Manpower shortage for HICC<br>➢ Paper based reporting system for HICC
**Biomedical Waste Management**	**Patient**	➢ Poor awareness about hazards of biomedical waste<br>➢ Patient crowding & littering inside hospital campus
	**Healthcare workers**	➢ Lack of motivation to attend BMWM training<br>➢ Non-compliance to standard guidelines<br>➢ Job insecurity among contractual workers<br>➢ Outsourced staff intake alcohol during duty hours
	**Hospital Administration**	➢ Irregular inspection for appropriate BMW disposal<br>➢ Lack of sewage treatment plant
	**Health System**	➢ Insufficient supply of BMW management materials (buckets, covers, closed bins, gloves, bags & masks)<br>➢ Challenge in solid waste management under municipality<br>➢ Lack of manpower to transport BMW to storage facility
**Blood Safety**	**Patient**	➢ Blood donors engage in high-risk behavior<br>➢ Professional donors selling blood for money
	**Healthcare workers**	➢ Poor logs and record maintenance by staffs
	**Hospital Administration**	➢ COVID-19 crisis led failure in blood donation camps and manpower training

	**Health System**	➢ Shortage of manpower for transfusion services<br>➢ Only blood storage facility available in some GHs<br>➢ Poor supply of blood transfusion materials
**Antimicrobial Stewardship**	**Patient**	➢ Self-medication with high level antibiotics<br>➢ OOPE due to unavailability of high-end antibiotics
	**Healthcare workers**	➢ Non-compliance to ICMR antibiotic guidelines<br>➢ Lack of awareness about prescription audits
	**Hospital Administration**	➢ Frequent stock-out of antimicrobials<br>➢ Lack of facilities to perform culture sensitivity<br>➢ No prescription audits
	**Health System**	➢ Non-availability of high-end antibiotics<br>➢ Antibiotic supply with a deadline to empty stocks
**COVID-19 Safety**	**Patient**	➢ Restless patients during triaging<br>➢ Visitors entering COVID-19 suspect/isolation ward<br>➢ COVID patients goes home against doctor's orders
	**Healthcare workers**	➢ Excess workload and hectic duty schedule
	**Hospital Administration**	➢ No dedicated staff for doing COVID-19 duty
	**Health System**	➢ Non-compliance to instructions from hospitals
**Medication Safety**	**Patient**	➢ Discontinuing medications following ADR
	**Healthcare workers**	➢ Unwillingness to comply with standard guidelines

		➢ Non-reporting of near expiry or expired drugs to hospital administration
	**Hospital Administration**	➢ Lack of awareness about ADR reporting in GHs
	**Health System**	➢ Delay or non-monitoring of ADR events
**Procedural and Device Safety**	**Patient**	➢ Obsession towards unnecessary injections & procedures
	**Healthcare workers**	➢ Unwillingness to comply with standard guidelines<br>➢ CSSD staffs unaware of job responsibilities
	**Hospital Administration**	➢ Lack of manpower in CSSD<br>➢ Shortage of equipment<br>➢ Delayed repair/servicing of the equipment<br>➢ Rotation of CSSD staffs after training<br>➢ Lack of infrastructure for CSSD activities
	**Health System**	➢ Non-availability of biomedical engineer
**Patient Safety Research**	**Healthcare workers**	➢ Lack of awareness about patient safety theme<br>➢ Lack of motivation to conduct research
	**Hospital Administration**	➢ Lack of administrative support<br>➢ Lack of facility or setup to conduct research

	**Health System**	➢ Scarcity of funds for conducting research

Theme: Suggestions to overcome the challenges in implementation of National Patient Safety Implementation Framework		
**Sub-themes**	**Categories**	**Codes**
**Structural System for Quality & Safety**	**Healthcare workers**	➢ Proper maintenance of records, registers, logs<br>➢ "Service with smile"
	**Hospital Administration**	➢ Working towards NQAS and NABH accreditation<br>➢ Conducting patient satisfactory survey<br>➢ Proper HMIS system<br>➢ Continuing Medical Education & Training<br>➢ Signage board with list of available services
	**Health System**	➢ Improving doctor-patient & nurse-patient ratio<br>➢ Filling vacancy & recruiting additional manpower<br>➢ CME/Workshops/Trainings on newer updates<br>➢ Permanentize trained contractual staffs<br>➢ Manpower to handle clerical works & data entry<br>➢ Carryover option for funding

		➢ Incentivization/increment for quality-related work<br>➢ Separate wing for each domain of patient safety<br>➢ Conflict management & stress management classes<br>➢ Making all the GHs into training centre<br>➢ Filling posts like Dean/MS/RMO with doctors having qualification & experience in hospital administration<br>➢ Addition of "patient safety" theme in curriculum
**Hospital Infection Control**	**Healthcare workers**	➢ Surveillance by team of HCWs on daily basis
	**Hospital Administration**	➢ Sustaining COVID-19 infection control measures<br>➢ Audiovisual demonstration of infection control practices in patient waiting area<br>➢ System based data handling<br>➢ Root cause analysis and brainstorming
	**Health System**	➢ District-level panel of experts for monitoring & supervision
	**Patient**	➢ Follow "no waste littering policy"

**Biomedical Waste Management**	**Healthcare workers**	➢ Awareness generation about hazards of BMW
	**Hospital Administration**	➢ Continuous training, encouragement, and support<br>➢ Exclusive staffs for biomedical waste management
	**Health System**	➢ Uninterrupted supply of BMW management materials
**Blood Safety**	**Patient**	➢ Patient representation in transfusion committee
	**Healthcare workers**	➢ Educating patients about voluntary blood donation
	**Hospital Administration**	➢ Acknowledgement & recognition of blood donors<br>➢ Ensuring Medico-legal safety for blood handlers
	**Health System**	➢ Recruitment of separate medical officer-in-charge
**Antimicrobial Stewardship**	**Patient**	➢ Trust in doctors
	**Healthcare workers**	➢ Pill count during patient follow-up
	**Hospital Administration**	➢ Directive to follow ICMR guidelines<br>➢ Regularly updating antibiotic formulary<br>➢ Regular training on antibiotic prescription
	**Health System**	➢ Digitally monitoring the antimicrobial usage<br>➢ Curriculum focusing on antimicrobial stewardship
**COVID-19 Safety**	**Patient**	➢ Patient testimonials
	**Health System**	➢ Recognition and appreciation of medical colleges/GHs
**Medication Safety**	**Patient**	➢ Health literacy
	**Healthcare workers**	➢ Educating patients about adverse drug reactions

		➢ Pharmacogenomic studies
	**Hospital Administration**	➢ Cross-checking high end antibiotic prescription<br>➢ Celebrating pharmacovigilance week<br>➢ Repeated training on handling ADR<br>➢ Conducting prescription audit<br>➢ Electronic drug ordering system
	**Health System**	➢ Reporting system/Surveillance of minor ADR<br>➢ Uninterrupted & sufficient antibiotic stocks<br>➢ Availability of high-end antibiotics<br>➢ Generic drug prescription by private practitioners
**Procedural and Device Safety**	**Hospital Administration**	➢ Standardized safe surgical checklist<br>➢ Proper calibration of BP apparatus<br>➢ Annual maintenance of equipment<br>➢ Clinical society meeting for doctors
	**Health System**	➢ Dedicated biomedical engineer<br>➢ Multi-storage & engineering support system for repairing/servicing equipment

**Patient Safety Research**	**Hospital Administration**	➢ Encouragement and support to faculties
	**Health System**	➢ Fund allocation/grants

Supplement Table S4: Best and Innovative Practices Across Surveyed Public Health Facilities in Tamil Nadu

Best /Innovative Practices
"Patient satisfactory survey"
"Patient representation in framing the blood safety policies of the facility"
"Utilization of WHO NET software for automated data entry and review in microbiological practices"
"Liaison with community organizations like Rotary & Lions Club for outreach activities like blood donation camps, awareness campaigns & drives"
"Dedicated staff for coordinating NQAS accreditation"<br>*"Root cause analysis for corrective and preventive actions"*
"Conducts research on questions related to patient safety theme"
"Mercury spill kit for accidental leakage from devices"
"Utilization of "10R" checklist for safe drug administration"<br>*"Fish bone diagram exploring the long waiting time in pharmacy depicted"*
"Core committee monitoring the activities of all the committees in hospital"

"Applying Dakshata checklist to assess staff competency in safe childbirth"<br><br>*"Daily surveillance of hand hygiene practices in the hospital"*
"Patient satisfactory survey"
"Antimicrobial stewardship committee"<br><br>*"Celebrating pharmacovigilance week"*<br><br>*"Utilization of HMIS version 2.0"*<br><br>*"First ICMR sponsored VRDL laboratory in South India"*
"Dedicated quality control team"<br><br>*"Repeated training for staffs in handling adverse drug reactions"*
